# Multifactorial Analysis of Influences on Quality of Life in Cancer Patients

**DOI:** 10.3390/nu16183207

**Published:** 2024-09-22

**Authors:** Elwira Gliwska, Dominika Głąbska, Zuzanna Zaczek, Jacek Sobocki, Dominika Guzek

**Affiliations:** 1 Department of Food Market and Consumer Research, Institute of Human Nutrition Sciences, Warsaw University of Life Sciences (WULS-SGGW), 159C Nowoursynowska Street, 02-776 Warsaw, Poland; dominika_guzek@sggw.edu.pl; 2 Cancer Epidemiology and Primary Prevention Department, Maria Sklodowska-Curie National Research Institute of Oncology, 15B Wawelska Street, 02-034 Warsaw, Poland; 3 Department of Dietetics, Institute of Human Nutrition Sciences, Warsaw University of Life Sciences (WULS-SGGW), 159C Nowoursynowska Street, 02-776 Warsaw, Poland; dominika_glabska@sggw.edu.pl; 4 Department of Human Nutrition, Faculty of Health Sciences, Medical University of Warsaw, 27 Erazma Ciolka Street, 01-445 Warsaw, Poland; zuzanna.zaczek@wum.edu.pl; 5 Department of General Surgery and Clinical Nutrition, Centre of Postgraduate Medical Education in Warsaw, 231 Czerniakowska Street, 00-416 Warsaw, Poland; jsobocki@mp.pl

**Keywords:** quality of life, cancer, enteral nutrition, EORTC QLQ-C30

## Abstract

Background/Objectives: The diminished quality of life experienced by cancer patients impacts not only the individuals but also the broader healthcare system. Despite the effect that cancer has on quality of life both during and after treatment, quality of life assessments are not yet commonly implemented, and the specific factors influencing quality of life are not well understood. The aim of this study was to conduct comprehensive multifactorial analysis to identify the factors that influence the quality of life in cancer patients. Methods: This study comprised a cohort of 108 patients diagnosed with cancer. The EORTC QLQ C-30 and EORTC QLQ-INFO 25 questionnaires were used to interview participants, along with questions related to disease course and lifestyle factors. Results: Differences were found in various subdomains among male versus female, individuals who solicited nutritional information in cancer themselves versus those who did not, individuals who reported consulting a dietitian versus those who did not, individuals who reported a recent decrease in their food intake versus those who did not report it. The results of the stepwise multiple regression show the following factors as the most important factors affecting their quality of life: patients soliciting nutritional information, reduced food intake, types of nutrition support, age, gender, seeking consultation with a dietitian, place of residence, and education level. Conclusions: In addition to sociodemographic variables and those related to the location, course, and treatment of cancer, including type of cancer, the variable related to patients’ declared need for soliciting nutritional information and seeking consultation with a dietitian may also influence quality of life. Therefore, the above factors in determining the quality of life and well-being of patients should also be considered in a holistic approach to patient care.

## 1. Introduction

As the share of older people in Europe increases, approximately 21% (~95 billion) of Europeans are over the age of 65 in 2020. This demographic shift toward an older population is substantial. This demographic segment, which constitutes one-fifth of the European population, is expected to account for over 62% of newly diagnosed cancer cases (equating to 1.7 million cases) and approximately 76% (amounting to 0.98 million) of total cancer-related deaths [1]. In turn, age is a recognized factor contributing to cancer risk, hence the anticipation of not only a higher incidence of cancer cases but also an increase in the number of cancer survivors. Given that cancer is increasingly viewed as a chronic condition, it is emphasized to assess not only treatment effectiveness but also the broader aspects of quality of life, which can have lasting impacts on the patient’s life after treatment [2]. The monitoring and enhancement of the quality of life among cancer patients are integral components of Europe’s Cancer Beating Plan—an initiative that highlights the importance for clinicians and healthcare professionals to consider not only life expectancy but how patients will experience their lives after treatment [3].

The impaired quality of life in cancer patients affects individuals, families, and society as a whole. Cancer‘s total cost was around EUR 199 billion in 2018 in EU-27 plus Iceland, Norway, Switzerland, and the United Kingdom, and this health expenditure related to cancer has increased faster than the increase in cancer incidence. Although productivity losses from premature mortality decreased from EUR 77 to EUR 70 billion between 1995 and 2018, largely due to fewer cancer-related deaths in the working-age population, the impact of health-related quality of life on work performance requires further investigation [4].

The World Health Organization (WHO) defines quality of life as an individual‘s subjective evaluation of their life circumstances within the cultural and value frameworks of their society, relative to their goals, expectations, standards, and concerns [5]. Therefore, quality of life is influenced by numerous factors, making it a multidimensional concept. Among cancer patients, several key domains are commonly disrupted, including physical, mental, and social aspects. Patients undergoing treatment often experience symptoms such as fatigue, insomnia, pain, and loss of appetite. Additionally, anxiety, stress, and depression are frequently observed [6]. Conversely, long-term impacts on quality of life include disturbances in social, role, emotional, cognitive, and physical functioning. Patients may also experience persistent symptoms like dyspnea. Moreover, the aftermath of cancer may lead to future financial challenges [7]. Some studies have indicated that cancer survivorship can be linked with co-morbid depression, anxiety, and symptoms of post-traumatic stress disorder (PTSD) [8,9,10].

During the COVID pandemic, cancer patients were particularly affected, which may have influenced their perception of the disease. The literature widely documents the adverse effects of COVID-19 on cancer patients and survivors, including increased psychological distress, disruptions in treatment schedules, heightened infection risk due to compromised immune systems, and challenges in accessing healthcare services [11,12,13,14,15,16,17]. These factors could have significantly altered the experiences and perspectives of individuals dealing with cancer during this unprecedented time, highlighting the need for tailored support and comprehensive care strategies to address their unique needs.

To obtain a clear and reliable understanding of cancer patients’ quality of life, it is essential to use standardized tools dedicated to cancer patients. The European Organization for Research and Treatment of Cancer Quality of Life Questionnaire Core 30 (EORTC QLQ-C30) is a widely recognized, standardized tool for assessing quality of life in cancer patients [18]. This questionnaire covers various domains such as the physical, role, emotional, cognitive, and social functioning, among others. Its comprehensive nature allows healthcare professionals to gain insights into the multifaceted impacts of cancer and its treatment on patients’ lives. Utilizing the EORTC QLQ-C30, healthcare providers can systematically evaluate patients’ quality of life, monitor changes over time, and customize interventions to address specific areas of concerns, thus facilitating patient-centered care and improving overall treatment outcomes [18].

To understand the influences on quality of life, it is important to consider specific clinical factors. Nutritional information and reduced food intake should be included in the assessment, as they may be related to treatment outcomes, as reduced food intake is linked to malnutrition. In particular, insight into cancer malnutrition can help in the design and implementation of oncology services to prevent poor patient outcomes [19]. Moreover, types of nutritional support and dietitian consultations reflect the proactive management of nutritional needs, which can improve patient well-being [20]. Additionally, sociodemographic factors such as age, gender, place of residence, and education level are often taken into consideration in clinical research because they have been identified as factors that influence the likelihood of malnutrition [19], and therefore, they can influence patient outcomes. Together, these factors could offer a comprehensive understanding of how various elements impact the quality of life in cancer patients, addressing key gaps in current research.

Despite growing awareness of cancer‘s impact on quality of life, current researches remain incomplete in key areas. It is crucial to thoroughly evaluate the factors influencing the severity and occurrence of factors affecting quality of life.. Identifying patient groups at higher risk of quality of life impairment and understanding avoidable factors that worsen quality of life appear to be crucial. Moreover, limited data exists on specific national contexts, such as Poland, where healthcare challenges may uniquely affect patient outcomes. This study addresses this gap with a multifactorial analysis of quality of life among Polish cancer patients, offering new insights in this area. Such research not only enhances understanding of the complexities surrounding cancer care but also enables the implementation of targeted support systems and interventions that can have a positive impact on the lives of those affected by cancer. These findings will contribute valuable data to better prepare for healthcare challenges and demographic shifts that are occurring. Therefore, the aim of this study was to conduct comprehensive multifactorial analysis to identify factors that influence quality of life in cancer patients. The findings aim to provide insights to guide targeted interventions and improve support systems to enhance the quality of life for cancer patients.

## 2. Materials and Methods

### 2.1. Ethical Statement

This study was conducted based on the guidelines of the Declaration of Helsinki. Each patient was informed of the purpose and nature of the research and provided their informed consent. The Bioethics Committee’s approval to conduct this study was obtained from the Bioethics Committee of the Medical Center for Postgraduate Education in Warsaw, Poland, on 14 July 2021, Order 116/2018.

### 2.2. Study Design and Setting

This cross-sectional study involved a cohort of patients diagnosed with cancer. Participants were recruited from two locations between January 2022 and September 2023: the Gastroenterology Unit at the Oncological Hospital in Warsaw, Poland, and the Polish Outpatient Clinic for Parenteral and Enteral Nutrition in Warsaw, Poland.

### 2.3. Study Population

The study sample included 108 patients diagnosed with cancer. Purposive sampling was used to select participants. Inclusion criteria were as follows: participants over 18 years of age, a confirmed cancer diagnosis, native Polish speakers, demonstrated linguistic and cognitive competence, and provided informed consent to participate. Exclusion criteria included participants who provided nonsensical or no information in the questionnaire.

### 2.4. Study Tools

Assessments were conducted weekly by a dietitian experienced in the field who consistently visited the hospital clinic. The measurement time points were set at weekly intervals throughout the study period. Each assessment was conducted at the same time each week to ensure consistency in data collection and include patient rotation in the ward. The overall duration of the data collection spanned from January 2022 to September 2023, during which the dietitian gathered data on quality of life and nutrition-related information from each eligible participant during every weekly visit. The EORTC QLQ-C30 questionnaire and EORTC QLQ-INFO 25 module were used to interview participants, along with questions related to disease course and lifestyle factors [21]. Information about the disease and its progression was verified in the patient‘s records.

The EORTC QLQ-C30 is a useful validated questionnaire [22], available in 122 official language versions [23]. The EORTC QLQ-INFO 25 questionnaire was administered with the EORTC QLQ-C30 and is a reliable and valid instrument [24], available in 33 official language versions [25].

Multi-item scales are used in the EORTC QLQ-C30 questionnaire, encompassing five functional scales (physical, role, cognitive, emotional, and social functioning), a global quality of life scale, and three symptom scales (fatigue, pain, and vomiting/nausea). Additionally, six individual symptom scales are included (dyspnoea, insomnia, loss of appetite, constipation, diarrhea, and financial difficulties). The standard version, Version 3.0, uses four-item scales for 28 questions and seven-item scales for assessing general health and quality of life over the past week. Responses range from 1 to 4 for the four-item scales and form 1 to 7 for the seven-item scales, which are then linearly transformed into a 0–100 scale. The EORTC QLQ-INFO 25 module specifically focuses on assessing the information needs of cancer patients, including satisfaction with information received, desire for more information, and perceived difficulty in understanding medical information. Using both the EORTC QLQ-C30 and EORTC QLQ-INFO 25 modules, this study aimed to provide a comprehensive understanding of not only the physical and psychological aspects of cancer patients’ quality of life but also their informational needs and experiences. This multifaceted approach allows for the exploration of the factors influencing patients’ overall well-being.

Moreover, an interview-based questionnaire, including both open-ended and closed-ended questions, was used to collect data on general sociodemographic characteristics. These included information on gender, age, educational level, place of residence, employment status, and economic situation (subjective assessment).

Weight loss was calculated based on current weight at clinic visits and self-reported weight six months before. Based on this, patients were categorized into those who had weight loss above and below 10%, as it has been indicated that weight loss of more than 10% in cancer patients is associated with deterioration in quality of life [26]. The Body Mass Index (BMI) was calculated based on weight (kg) divided by height (m) squared.

The nutritional status of each patient was evaluated by a qualified clinical dietitian using the Subjective Global Assessment (SGA) questionnaire results provided by a healthcare professional at the clinic. This evaluation also considered additional factors, including unintentional weight loss, reduced food intake, and disease progression, as documented in patient records and captured through questionnaire responses. The dietitian systematically reviewed these elements to form a comprehensive picture of the patient’s nutritional health. The process involved correlating data from the SGA with clinical observations and patient-reported information to ensure a thorough assessment.

### 2.5. Statistical Analysis

The Shapiro–Wilk test was applied to assess the normality of the distribution. Group comparisons were conducted using the chi-squared test, Student’s *t*-test for parametric distributions, and the Mann–Whitney U test for nonparametric distributions. The internal consistency of the EORTC QLQ-C30 scales was evaluated using Cronbach’s alpha coefficient, with a value of ≥0.70 considered as acceptable [27].

Stepwise multiple regression analysis was conducted, with the following variables: age, gender, education level, employment, place of residence, type of cancer, weight loss over 10%, BMI, economic situation, reduced food intake, patients soliciting nutritional information, types of nutrition support, and seeking consultation with a dietitian. The analysis reported both unstandardized and standardized β-coefficients accompanied by *p*-values. A significance level of *p* ≤ 0.05 was considered statistically significant. The statistical analysis was performed using Statistica 13.3 (StatSoft Inc., Tulsa, OK, USA).

## 3. Results

The sociodemographic characteristics of the patients are presented in Table 1. The median age of the study group was 60 years old, with BMI values interpreted as normal body mass in majority of the studied group, and approximately 10% weight loss observed recently. Over half of patients were women, with a predominant share of a higher education level, and nearly half of patients reported being retired. Around 30% of individuals lived in rural areas, as well as around 30% lived in large cities of over 500,000 inhabitants. Half of the patients reported reduced food consumption, and the majority of them solicited nutritional information related to their health status.

Cancer sites in the study group are presented in Table 2. More than half of the patients were diagnosed with head and neck cancer.

The results of the five functional scales, global quality of life scale, three symptom scales, and six individual symptom scales of EORTC QLQ-C30 are presented in Table 3. Cronbach’s alpha values for the individual scales ranged from fair (cognitive in functional scales) to very good (pain in symptom scales), indicating the reliability of this tool and the results obtained through it.

Table 4 presents the mean and median scores of the five functional scales, global quality of life scale, three symptom scales, and six individual symptom scales of the EORTC QLQ-C30, stratified by the gender of the analyzed group. Statistically significant differences were found in global health status and the cognitive, emotional, social subdomains (functional scale), as well as for the fatigue subdomain (symptom scale) between genders. Male patients scored higher than female patients in the following measures: the cognitive, emotional, and social subdomains and global health status. Female patients scored higher than male patients only in the fatigue subdomain. In the case of the physical subdomain (functional scale), a difference close to statistical significance was observed, and a tendency to higher scores in male patients may be indicated.

Table 5 presents the results of the five functional scales, global quality of life scale, three symptom scales, and six individual symptom scales of the EORTC QLQ-C30, stratified by the reported recent decrease in the food intake. Statistically significant differences were observed in global health status, the cognitive and emotional subdomains (functional scales), as well as for the pain (symptom scale), insomnia, and appetite loss subdomains (single-item measures) between individuals who reported a recent decrease in their food intake and those who did not report it. Individuals who did not report a recent decrease in their food intake scored higher than those who reported it in the cognitive and emotional subdomains, as well as in global health status. However, individuals who reported a recent decrease in their food intake scored higher in the pain (symptom scale), insomnia, and appetite loss subdomains (single-item measures) than those who did not report a reduced food intake.

Table 6 presents the results of the five functional scales, global quality of life scale, three symptom scales, and six individual symptom scales of the EORTC QLQ-C30, stratified by reporting to have consulted a dietitian. Statistically significant differences were found in the role and cognitive subdomains (functional scales), as well as for the fatigue (symptom scale) and appetite loss subdomain (single-item measure) between individuals who reported consulting a dietitian and those who did not. Individuals who did not report consulting a dietitian scored higher in the role and cognitive subdomains (functional scales), while individuals who reported consulting a dietitian scored higher in the fatigue (symptom scale) and appetite loss subdomains (single-item measure) than individuals who reported consulting a dietitian. In the case of the social subdomain (functional scale) and global health status, higher scores were observed for individuals who did not report consulting a dietitian than those who reported it, while for the pain subdomain (symptom scale), higher scores were observed for individuals who reported consulting a dietitian than those who did not. However, it should be noted that these latter relationships only indicate a trend (level close to the statistical significance).

In Table 7, the results of the five functional scales, global quality of life scale, three symptom scales, and six individual symptom scales of the EORTC QLQ-C30 are presented, stratified by having declared to have solicited nutritional information. Statistically significant differences were found in all functional scales (physical, role, cognitive, emotional, and social subdomains), in two out of three subdomains on the symptom scales (fatigue and pain subdomains), and in the insomnia subdomain (single-item measure), as well as in global health status between individuals who solicited nutritional information and those who did not. Individuals who did not solicit nutritional information scored higher in the physical, role, cognitive, emotional, and social subdomains (functional scales) than individuals who solicited nutritional information, whereas individuals who solicited nutritional information scored higher in the fatigue and pain subdomains (symptom scales), insomnia subdomain (single-item measure), and global health status than individuals who did not solicit nutritional information.

In Supplementary Table S1, the results of the five functional scales, global quality of life scale, three symptom scales, and six individual symptom scales of the EORTC QLQ-C30 are presented, stratified by patient education level. In Supplementary Table S2, the results of the five functional scales, global quality of life scale, three symptom scales, and six individual symptom scales of the EORTC QLQ-C30 are presented, stratified by place of residence. The analysis was conducted in larger subgroups due to their size.

**Figure 1:**
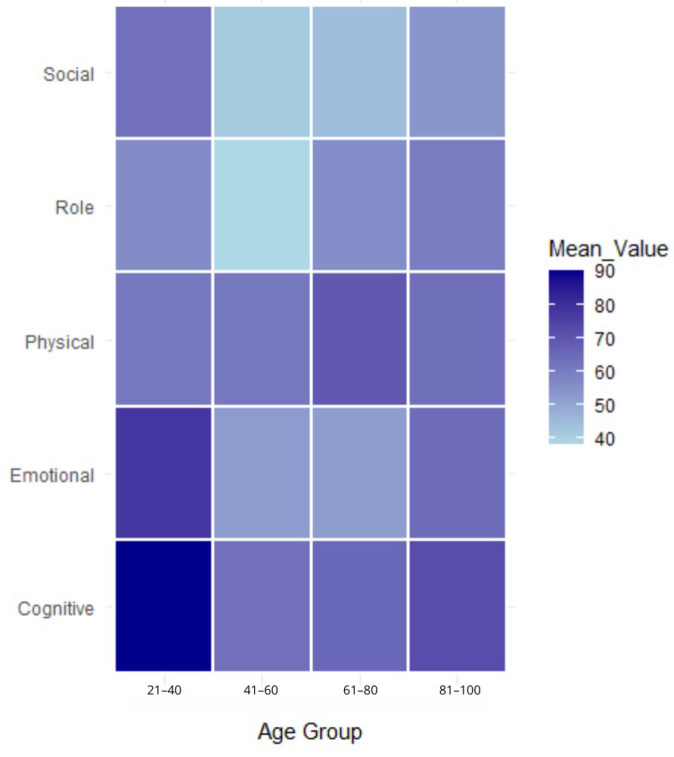
Heatmap of mean response score by age group.

Figure 1 presents a heatmap of the mean response score by age group. The heatmap visualizes the mean value across a spatial grid. Density scores were computed based on the number of data points assigned to each grid cell, with more intensive colors indicating higher densities, visualizing the number of points in each scale for each age group. Differences in the quality of life domains across various age groups can be observed.

**Figure 2:**
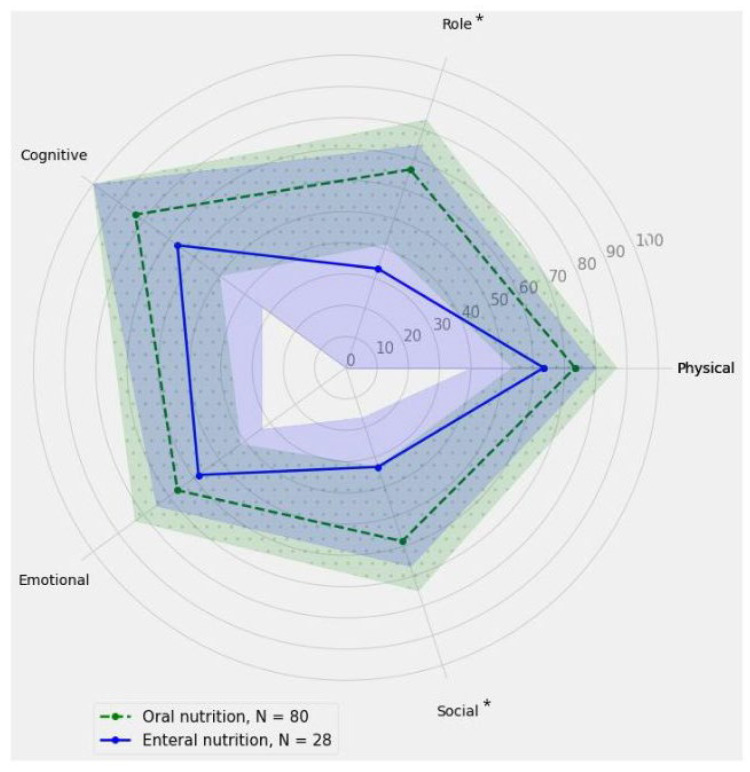
The comparison of the main subscales’ measurements of the EORTC QLQ-C30 for orally and enterally fed patients in the study group. The solid line represents the results obtained in points for individual subscales within the enteral nutrition group, while the dashed line corresponds to the results obtained in points for individual subscales within the oral nutrition group. The shaded areas indicate the confidence intervals (CIs). An asterisk indicates statistical significance at the level of *p* ≤ 0.05.

Figure 2 shows a comparison of the main subscales’ measurements of the EORTC QLQ-C30 for orally and enterally fed patients in the study group. As seen on Figure 2, enterally fed patients achieved lower scores than orally fed patients for the role and social subdomains, as well as for general health status in the EORTC QLQ-C30. Simultaneously, enterally fed patients achieved higher scores than orally fed patients for the vomiting/nausea (*p* = 0.0308), dyspnea (*p* = 0.0306), and appetite loss subdomains (*p* = 0.0004) on the EORTC QLQ-C30 questionnaire.

**Figure 3:**
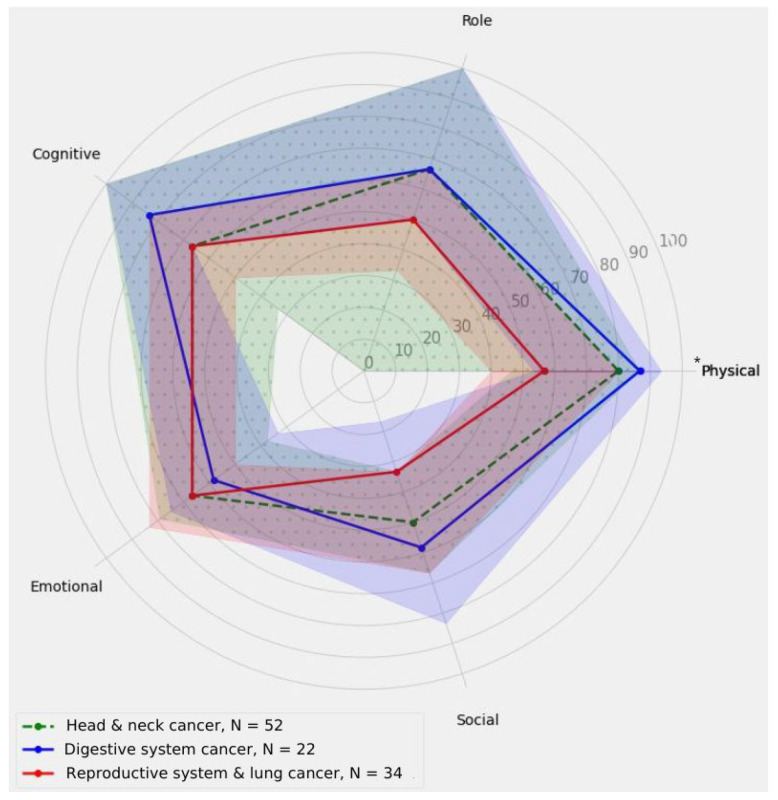
The comparison of the main subscales’ measurements of the EORTC QLQ-C30 for different type of cancer groups. The solid blue line represents the results obtained in points for individual subscales within digestive system cancer patients, the solid red line represents the results obtained in points for individual subscales within reproductive system cancer patients, and the dashed line corresponds to the results obtained in points for individual subscales within the head and neck cancer patient group. The shaded areas indicate the confidence intervals (CIs). An asterisk indicates statistical significance at the level of *p* ≤ 0.05.

Figure 3 shows a comparison of the main subscales’ measurements of the EORTC QLQ-C30 for different type of cancer groups. As shown in Figure 3, there is significant variation in the physical subdomain depending on different cancer location groups. Patients with reproductive system and lung cancers achieved lower scores than those with digestive system cancer, whereas digestive system cancer patients achieved similar scores in the physical subdomain as head and neck cancer patients in a post hoc analysis (*p* = 0.0347). This indicates the need for a specific approach to quality of life and its various domains depending on the type of cancer.

The results of the stepwise multiple regression analysis for the EORTC QLQ-C30 scale in a model that included age, gender, education level, employment status, place of residence, type of cancer, weight loss, over 10% weight loss, BMI, economic situation, reduced food intake, patients soliciting nutritional information, types of nutrition support, and reported to have consulted a dietitian as variables are presented in Supplementary Tables S1–S19, showing the results within each scale and measure. The analysis revealed that, depending on the scale of the EORTC QLQ-C30, various factors influenced the scores obtained. These factors included variables such as patients soliciting nutritional information, reduced food intake, types of nutrition support, age, gender, seeking consultation with a dietitian, place of residence, and education level.

An additional stepwise multiple regression analysis for the global health status (QoL) of the EORTC QLQ-C30 in a model with five functional scales (physical, role, cognitive, emotional, and social) and three symptom scales (fatigue, pain, and nausea/vomiting) is presented in Table 8. This analysis revealed that functional scales (physical and social subdomains) and appetite loss (single-items measures) had the greatest influence on the global health status (QoL).

In summary, the results of the stepwise multiple regression indicate that the following factors are the most important affecting the quality of life in a group of patients with various types of cancer: patients soliciting nutritional information, reduced food intake, types of nutrition support, age, gender, seeking consultation with a dietitian, place of residence, and education level.

## 4. Discussion

This study investigated various factors influencing quality of life among cancer patients, indicating the impact of patients soliciting nutritional information, reduced food intake, types of nutrition support, age, gender, seeking consultation with a dietitian, place of residence, and education level on quality of life aspects in cancer patients.

It is also important to understand the patient‘s attitude toward nutrition and their efforts to seek nutrition information, including their need for knowledge about the appropriate diet to follow during cancer treatment. Based on the multifactorial analysis, it was found that among the most important factors contributing to the varied outcomes in different domains of quality of life are soliciting nutritional information, reduced food intake, types of nutrition support, seeking consultation with a dietitian, and some sociodemographic variables (such as age, gender place of residence, and education level).

### 4.1. Quality of Life and Soliciting Nutritional Information

The presented study found that patients who did not actively seek information on nutrition tended to report higher scores across various functional domains, including physical, role, cognitive, emotional, and social functioning. This suggests that while seeking nutrition information may reflect underlying health challenges or higher anxiety levels, it could also indicate proactive patient engagement in managing their condition, as discussed in studies by Cook et al. [28] and Kenny et al. [29]. Despite the fact that nutritional information during cancer treatment may result in improved weight maintenance and energy intake, an overload of information received (or obtained from the internet) may lower the quality of life, especially for patients with low cognitive abilities. This excess information could be overwhelming or unclear, making it difficult for patients to remember and understand. In a study by Eraslan and Tufan [30], it was stated that patients’ searches for information about their treatments on the internet may cause cancer information overload (CIO), depression, and anxiety. The results of Posma et al.’s [31] study also indicate that patient education should be more tailored to specific needs of older cancer patients, given the decline in cognitive, vision, and hearing functions with age.

### 4.2. Quality of Life and Reduced Food Intake

Nutrition emerged as a critical determinant of QoL in this study. A significant number of patients experienced recent weight loss. This underscores the nutritional challenges and the need for intervention strategies to address this issue. Patients who had a recently reduced food intake exhibited poorer cognitive and emotional functioning and increased pain, insomnia, and appetite loss compared to those who did not experience such a reduction. This highlights the importance of proper food intake and interventions in enhancing patients’ overall well-being, consistent with findings from Keaver et al. [32] and Takata et al. [33].

### 4.3. Quality of Life and Types of Nutrition Support

While enteral nutrition is necessary for patients who cannot tolerate oral intake, the data from this study consistently suggest that oral nutrition is associated with better quality of life outcomes across multiple domains, including the role and social subdomains, as well as for general health status. This stays in line with results from Donohoe et al. [34] and Dashti et al. [35]. Especially notable is that Donohoe et al. [34] also indicated that in the majority of patients, despite supplemental nutrition, weight loss occurs, which may negatively impact the quality of life. Patients receiving enteral nutrition have more severe illnesses, including advanced cancer and higher rates of malnutrition, they more often have a more serious medical condition. They may also undergo more extensive medical treatments and procedures, which can contribute to poorer overall health and well-being outcomes compared to those who can maintain oral nutrition. However, patients relying on enteral feeding often encounter challenges that can negatively impact their quality of life. These challenges include physical discomfort from tube placement and maintenance, activity restrictions due to feeding schedules or mobility issues, and potential complications like infections or gastrointestinal discomfort. However, it should be notice that enteral nutrition has the best risk-benefit ratio for malnourished surgical cancer patients [36]. Considering this factors, lower scores of quality of life can be observed in patients receiving enteral nutrition due to additional factors.

### 4.4. Quality of Life and Age

Age was found to be a significant factor influencing quality of life outcomes in the study group. While the study did not show a significant impact of age on overall quality of life or functional scales, older patients consistently reported higher scores in specific symptom-related areas such as insomnia and diarrhea. This indicates that age may play a role in certain symptoms experienced by cancer patients, although the effect sizes were modest. This finding aligns with previous research by Shrestha et al. [37], which emphasizes that advancing age correlates with diminished QoL parameters in cancer patients. In Ligt et al. study [38], it was found that older age significantly influences deterioration in the physical functioning scores but improves emotional functioning scores. In this study, it was observed that higher cognitive functioning scores were found in younger patients. However, it should be noted that the study group was generally older (median age was 60.0 years) than in Ligt et al. [38] study (mean age was 54.0 years). The implications suggest that interventions tailored to address age-related challenges could potentially enhance overall QoL among older cancer patients. Effective care for elderly cancer patients should prioritize these domains that are most influenced.

### 4.5. Quality of Life and Gender

Across various domains of quality of life assessed by the EORTC QLQ-C30 scale, being male generally was associated with better functional scales scores (cognitive, emotional, and social subdomains) and global health status as quality of life outcomes. These findings suggest that gender differences may influence how cancer patients perceive and experience their quality of life, with males potentially experiencing better cognitive, emotional, social functioning, lower fatigue levels, and better overall quality of life compared to females scoring higher in fatigue related scale in this study population. Similar gender differences have been documented in studies by Tarar et al. [39] and Jehn et al. [40], highlighting those tailored approaches in cancer care, sensitive to gender-specific needs and challenges, are essential for optimizing QoL outcomes. Nevertheless, it should be noted that while the impact of gender varies across the EORTC QLQ-C30 scales as reported in the conducted study, the influence of health conditions remains highly significant. These studies collectively emphasize the importance of a gender-sensitive approach in cancer care, recognizing the distinct needs and challenges faced by men and women. Addressing these differences can improve quality of life outcomes and provide more effective support for cancer patients.

### 4.6. Quality of Life and Seeking Consultation with a Dietitian

This study highlights a significant association between consulting a dietitian and better scores in several domains related to quality of life (QoL). Specifically, patients who sought dietary consultation experienced more fatigue, pain, and insomnia, but reported a better overall health perception. Simultaneously, individuals who did not seek nutritional information had higher scores on the functional scales, which assess physical, role, cognitive, emotional, and social functioning. This observation aligns with the broader literature, which suggests that nutritional counseling can play a significant role in managing both physical and psychological aspects of health in cancer patients [32,33,34,35,36,37,38,39,40,41,42,43]. Despite their awareness of the need for dietary consultations, cancer patients may still face increased stress and information overload [44]. Especially considering the fact that cancer patients experience some impact of the disease on their food intake, which likely includes the dietary recommendations provided by a dietitian. These psychological aspects affecting food consumption may be linked with behavioral responses such as learned food aversions, changes in food preferences, and anticipatory nausea and vomiting [45]. This may contribute to a deeper sense of being overwhelmed by conflicting messages. Consequently, this could result in lower scores in the cognitive scale (especially concerning questions like: ‘Did you have difficulties remembering?’). It should be noted that feeling overwhelmed by conflicting information may not stem from a single source [44]. However, the availability of nutritional counseling remains a critical issue. In Poland, despite the recognized benefits, nutritional counseling services are not sufficiently widespread to meet the needs of all cancer patients. Hospitals employ a relatively small number of dietitians, and the estimated duration of a dietetic consultation, including the measurement of lean body mass and a nutritional interview, is just 30 min [46]. This limitation raises concerns about the accessibility and equity of nutritional care within the healthcare system. It appears that patients who seek out nutritional counseling independently may be those who are either particularly motivated or facing more severe health challenges. This situation reflect a disparity where only a subset of patients benefits from nutritional guidance, which is reflected in a studied group within association between seeking consulting a dietitian and reduced cognitive function, as it may be supposed, that patients with reduced cognitive function may need additional support [47].

Taking into account the need for deepen dietary counseling, the problem is associated with inadequate support in this area. Sullivan et al. [48] reported that only a small fraction of cancer survivors had access to a registered dietitian. Many survivors, faced conflicting nutrition information from various sources, turned to alternative dietary strategies or engaged in high levels of food avoidance. This confusion highlights the critical need for reliable and consistent nutritional guidance, which is currently lacking in many cancer care settings. Similar observations are made by Trujillo et al. [49], who states that lack of access to nutrition care in outpatient cancer centers is a critical issue in the U.S. healthcare system. Furthermore, van Dongen et al. [50] highlighted the increasing expectation, both from formal and informal caregivers, as well as from cancer patients themselves, to self-manage their diets. Their findings also indicated that a significant proportion of patients—70–82%—adopted balanced diets, which were linked to improvements in QoL, increased strength, and better symptom management [50]. This study supports the benefits of consulting a dietitian, but also highlights systemic challenges. The disparity in access to nutritional counseling services indicates that broader implementation and integration of these services within healthcare systems are essential. Future research should explore strategies to increase the availability and accessibility of nutritional counseling to ensure that all cancer patients can benefit from these critical support services.

### 4.7. Quality of Life and Place of Residence

The findings of the presented study suggest that the place of residence may influence declared symptoms related to quality of life, such as insomnia. However, in this study, this variable had a significant impact only when combined with other variables in a multivariate analysis characterizing the group. Nevertheless, it should be noted that the primary impact of residence is likely on access to medical care and support services. A systematic review by Afshar et al. [51] indicates that, in most studies, rural cancer patients often experience worse survival outcomes compared to their urban counterparts, potentially due to urban-rural disparities in healthcare access. These disparities may also affect the severity of symptoms in cancer patients. Additionally, the location of residence can impact the availability of support services, such as nutritional counseling and psychological support. Payne et al. [52] highlight that patients residing far from hospitals may experience increased emotional and psychological distress during hospital stays, which can exacerbate symptoms. Furthermore, the strength of social networks and community support can vary by location as stated by Carnahan at al. [53]. Patients in close-knit communities may receive greater emotional support and practical assistance, which can positively influence their quality of life [51]. However the relationship between place of residence and quality of life requires further research.

### 4.8. Quality of Life and Education Level

The presented research suggests that the educational level of cancer patients may significantly impact their QoL, particularly in domains such as physical and cognitive functioning as well as in the fatigue domain. Higher educational levels are correlated with better scores in the fatigue subdomain, but not on the physical and cognitive functioning scales. Konieczny et al. [54] found that higher QoL—measured by functional scales and symptom intensity—was, among other factors, linked to higher education level, and a favorable financial situation. Higher education might be associated with higher income, contributing to better well-being and a greater sense of security. Better-educated patients may also have a better understanding of their medical situation and procedures, which could influence QoL. Moreover, Fiscella et al. [55] found that patients with lower levels of education received slightly less time for answering their questions, exercise counseling, assessing their health knowledge, and negotiating. In terms of fatigue, it is worth noting that Avancini et al. [56] reported that cancer patients with a higher education level showed a greater willingness to engage in physical exercise. This increased motivation for exercise may contribute to be more physically fit and become tired less easily. This finding is consistent with the hypothesis that higher education is associated with improved health literacy and greater adherence to medical and nutritional advice, as evidenced by Nilsen et al. [57] in their study on cancer survivors. In conclusion, the level of education appears to have a meaningful impact on various dimensions of QoL in cancer patients, potentially through its influence on physical health, symptom management, and the adherence to health recommendations.

### 4.9. Study Limitations

This study has several limitations, including a small sample size, its focus on a single geographic region and clinic selection, a cross-sectional design preventing causal conclusions, and potential reporting bias. Future research should aim to address these limitations to more comprehensively understand the factors influencing the quality of life of cancer patients.

## 5. Conclusions

This study identifies differences in quality of life and symptom management across different patient sociodemographic and health behaviors, highlighting the importance of personalized nutritional and psychological support in improving patient outcomes. Univariate and multivariate analysis indicated that, in addition to sociodemographic variables and those related to the location, course, and treatment of cancer, including types of cancer, variables related to patients’ declared need for soliciting nutritional information and seeking consultation with a dietitian may also influence quality of life. While seeking nutrition information may reflect underlying health challenges, an excess of information could be overwhelming, making it difficult for patients to remember and understand. Therefore, these factors influencing the quality of life and well-being of patients should be integrated into a holistic approach to patient care. Healthcare providers should incorporate nutritional counseling tailored to cognitive abilities as part of patient care to enhance both quality of life and patient outcomes. The findings of this study could further enhance our understanding of the quality of life in cancer patients.

## Figures and Tables

**Table 1:** Sociodemographic characteristics of patients.

	Characteristics		Number (%)
Gender	Woman		58 (53.7%)
	Man		50 (46.29%)
Education level	Higher		49 (45.37%)
	General secondary		36 (33.33%)
	Vocational secondary		22 (20.37%)
	Primary		1 (0.93%)
Employment	Full-time permanent		28 (25.93%)
	Part-time permanent		4 (3.70%)
	Full- or parti-time temporary		5 (4.63%)
	Pension		7 (6.48%)
	Retirement		50 (46.30%)
	Not employed		14 (12.96%)
Place of residence	Rural area		36 (33.33%)
	Town, up to 20,000 inhabitants		14 (12.96%)
	City, 20,000 to 100,000 inhabitants		21 (19.44%)
	City, 100,000 to 500,000 inhabitants		5 (4.63%)
	City, over 500,000 inhabitants		32 (29.63%)
>10% weight loss	Yes		57 (52.78%)
	No		51 (47.22%)
Economic situation	Good		25 (23.15%)
	Rather good		40 (37.04%)
	Bad		20 (18.52%)
	Rather bad		10 (9.26%)
	Difficult to say		13 (12.04%)
Reduced food intake	Yes		60 (55.56%)
	No		48 (44.44%)
Patients soliciting nutritional information	Yes		71 (68%)
	No		33 (31%)
Types of nutrition support	Oral nutrition		80 (74.07%)
	Enteral nutrition		28 (25.92%)
Seeking consultation with a dietitian	Yes		67 (62.04)
	No		41 (37.96%)
Visited a dietitian	Yes		67 (62.03%)
	No		41 (37.96%)
	Mean ± SD	95% CI	Median * (IQR)
Age	58.20 ± 14.38	55.44–60.97	60 (21.75)
% Weight loss	9.52 ± 8.84	7.82–11.23	9.95 (14.73)
BMI	24.31 ± 4.82	23.38–25.24	23.49 (7.28)

* Non-distribution based on the Shapiro–Wilk test (*p* < 0.05); BMI—Body Mass Index.

**Table 2:** Cancer sites in the study group.

Cancer Site According to ICD 10	Name	Number (%)
C04	Malignant neoplasm of floor of mouth	10 (9.26)
C14	Malignant neoplasm of other and ill-defined sites in the lip, oral cavity, and pharynx	16 (14.81)
C15	Malignant neoplasm of esophagus	10 (9.26)
C16	Malignant neoplasm of stomach	5 (4.63)
C17	Malignant neoplasm of small intestine	1 (0.93)
C18	Malignant neoplasm of colon	4 (3.70)
C22	Malignant neoplasm of liver and intrahepatic bile ducts	4 (3.70)
C24	Malignant neoplasm of other and unspecified parts of biliary tract	1 (0.93)
C25	Malignant neoplasm of pancreas	3 (2.78)
C32	Malignant neoplasm of larynx	20 (18.52)
C34	Malignant neoplasm of bronchus and lung	4 (3.70)
C50	Malignant neoplasm of breast	10 (9.26)
C54	Malignant neoplasm of corpus uteri	1 (0.93)
C56	Malignant neoplasm of ovary	11 (10.18)
C61	Malignant neoplasm of prostate	8 (7.41)

**Table 3:** Mean and median scores of the five functional scales, global quality of life scale, three symptom scales, and six individual symptom scales of the EORTC QLQ-C30.

Measures	Subdomain	Mean Score ± SD	Median (IQR) *	95%CI	Cronbach’s Alpha Coefficient
Functional scales	Physical	65.56 ± 26.09	73.33 (40.0)	60.58–70.53	0.82
	Role	56.33 ± 34.39	66.67 (50.0)	49.77–62.89	0.86
	Cognitive	69.6 ± 27.70	75.0 (50.0)	64.31–74.88	0.51
	Emotional	59.34 ± 28.09	66.67 (37.5)	53.98–64.69	0.66
	Social	49.54 ± 30.95	50.0 (33.33)	43.63–55.44	0.71
Global health status		44.68 ± 21.36	41.67 (29.17)	40.60–48.75	-
Symptom scales	Fatigue	48.46 ± 29.20	44.44 (33.33)	42.89–54.03	0.70
	Pain	42.90 ± 34.35	33.33 (58.33)	36.35–49.45	0.88
	Vomiting/nausea	10.34 ± 17.16	0.0 (16.67)	7.07–13.61	0.58
Single-item measures	Dyspnea	19.14 ± 29.25	0.0 (33.33)	13.56–24.72	-
	Insomnia	46.30 ± 33.75	33.33 (33.33)	39.86–52.73	-
	Appetite loss	27.16 ± 31.62	33.33 (33.33)	21.13–33.19	-
	Constipation	24.38 ± 31.11	0 (33.33)	18.45–30.32	-
	Diarrhea	17.90 ± 30.38	0 (33.33)	12.11–23.70	-
	Financial difficulties	35.49 ± 35.38	33.33 (66.67)	28.74–42.24	-

* Non-distribution based on the Shapiro–Wilk test (*p* < 0.05).

**Table 4:** Mean and median scores of the five functional scales, global quality of life scale, three symptom scales, and six individual symptom scales of the EORTC QLQ-C30, stratified by the gender of the analyzed group.

Measures	Subdomain	Female (*n* = 58)			Male (*n* = 50)			*p*-Value
		Mean Score ± SD	Median (IQR)	95%CI	Mean Score ± SD	Median (IQR)	95%CI	
Functional scales	Physical	62.53 ± 24.43	63.33 (46.67) *	40–86.67	69.07 ± 27.73	80 (40) *	53.33–93.33	0.0836
	Role	52.59 ± 33.16	66.67 (33.33) *	33.33–66.67	60.67 ± 35.6	66.67 (66.67) *	33.33–100	0.1974
	Cognitive	61.78 ± 28.1	66.67 (50) *	33.33–83.33	78.67 ± 24.52	83.33 (33.33) *	66.67–100	0.0011
	Emotional	50.29 ± 28.57	50 (33.33)	33.33–66.67	69.83 ± 23.74	75 (33.33) *	58.33–91.67	0.0002
	Social	43.68 ± 30.08	33.33 (50) *	16.67–66.67	56.33 ± 30.84	66.67 (50) *	33.33–83.33	0.0267
Global health status		38.51 ± 18.59	33.33 (25) *	25–50	51.83 ± 22.29	50 (33.33)	33.33–66.67	0.0029
Symptom scales	Fatigue	56.90 ± 28.16	55.56 (44.44) *	33.33–77.78	38.67 ± 27.51	33.33 (33.33) *	22.22–55.56	0.0008
	Pain	45.69 ± 34.42	33.33 (66.67) *	16.67–83.33	39.67 ± 34.32	33.33 (66.67) *	0–66.67	0.3686
	Vomiting/ nausea	10.63 ± 17.85	0 (16.67) *	0–16.67	10 ± 16.5	0 (16.67) *	0–16.67	0.8703
Single-item measures	Dyspnea	22.41 ± 32.07	0 (33.33) *	0–33.33	15.33 ± 25.39	0 (33.33) *	0–33.33	0.2695
	Insomnia	51.15 ± 33.75	50 (33.33) *	33.33–66.67	40.67 ± 33.19	33.33 (66.67) *	0–66.67	0.1109
	Appetite loss	28.16 ± 31.72	33.33 (33.33) *	0–33.33	26 ± 31.79	16.67 (33.33) *	0–33.33	0.6607
	Constipation	22.41 ± 30.2	0 (33.33) *	0–33.33	26.67 ± 32.3	16.67 (33.33) *	0–33.33	0.4562
	Diarrhea	20.69 ± 32.34	0 (33.33) *	0–33.33	14.67 ± 27.9	0 (33.33) *	0–33.33	0.3182
	Financial difficulties	40.8 ± 38.51	33.33 (66.67) *	0–66.67	29.33 ± 30.6	33.33 (33.33) *	0–33.33	0.1626

* Non-distribution based on the Shapiro–Wilk test (*p* < 0.05).

**Table 5:** Results of the five functional scales, global quality of life scale, three symptom scales, and six individual symptom scales of the EORTC QLQ-C30, stratified by the reported recent decrease in food intake.

Measures	Subdomain	Group 1 (*n* = 60)			Group 2 (*n* = 48)			*p*-Value
		Mean Score ± SD	Median (IQR)	95%CI	Mean Score ± SD	Median (IQR)	95%CI	
Functional scales	Physical	63 ± 25.41	66.67 (43.33) *	43.33–86.67	68.75 ± 26.85	80 (33.33) *	53.33–86.67	0.1488
	Role	55 ± 34.62	66.67 (50) *	33.33–83.33	57.99 ± 34.38	66.67 (50) *	33.33–83.33	0.6340
	Cognitive	60.56 ± 27.61	66.67 (50) *	33.33–83.33	80.9 ± 23.57	83.33 (33.33) *	66.67–100	0.0001
	Emotional	54.17 ± 28.48	58.33 (41.67) *	33.33–75	65.8 ± 26.48	66.67 (25) *	58.33–83.33	0.0236
	Social	46.11 ± 32.09	41.67 (50) *	16.67–66.67	53.82 ± 29.22	66.67 (41.67) *	33.33–75	0.1848
Global health status		40.42 ± 20.23	41.67 (25)	25–50	50 ± 21.74	50 (33.33)	33.33–66.67	0.0198
Symptom scales	Fatigue	51.3 ± 27.38	44.44 (33.33) *	33.33–66.67	44.91 ± 31.26	38.89 (44.44) *	22.22–66.67	0.2140
	Pain	49.17 ± 32.68	33.33 (50) *	33.33–83.33	35.07 ± 35.1	33.33 (50) *	0–50	0.0324
	Vomiting/nausea	12.22 ± 18.38	0 (16.67) *	0–16.67	7.99 ± 15.37	0 (16.67) *	0–16.67	0.3553
Single-item measures	Dyspnea	23.89 ± 32.53	0 (33.33) *	0–33.33	13.19 ± 23.56	0 (33.33) *	0–33.33	0.1378
	Insomnia	53.33 ± 34.83	66.67 (33.33) *	33.33–66.67	37.5 ± 30.46	33.33 (66.67) *	0–66.67	0.0204
	Appetite loss	37.78 ± 33.88	33.33 (66.67) *	0–66.67	13.89 ± 22.63	0 (33.33) *	0–33.33	0.0002
	Constipation	25.56 ± 32.11	0 (33.33) *	0–33.33	22.92 ± 30.1	0 (33.33) *	0–33.33	0.7338
	Diarrhea	18.33 ± 31.55	0 (33.33) *	0–33.33	17.36 ± 29.16	0 (33.33) *	0–33.33	0.9630
	Financial difficulties	37.22 ± 36.36	33.33 (66.67) *	0–66.67	33.33 ± 34.38	33.33 (33.33) *	0–33.33	0.6362

* Non-distribution based on Shapiro–Wilk test (*p* < 0.05); Group 1—individuals reported recent decrease in food intake; Group 2—individuals who did not report recent decrease in food intake.

**Table 6:** Results of the five functional scales, global quality of life scale, three symptom scales, and six individual symptom scales of the EORTC QLQ-C30, stratified by reported consultation with a dietitian.

Measures	Subdomain	Group 1 (*n* = 67)			Group 2 (*n* = 41)			*p*-Value
		Mean Score ± SD	Median (IQR)	95%CI	Mean Score ± SD	Median (IQR)	95%CI	
Functional scales	Physical	64.58 ± 25.56	73.33 (40) *	46.67–86.67	67.15 ± 27.18	73.33 (53.33) *	40–93.33	0.4783
	Role	49 ± 36.22	50 (66.67) *	16.67–83.33	68.29 ± 27.59	66.67 (50) *	50–100	0.0087
	Cognitive	64.43 ± 29.86	66.67 (66.67) *	33.33–100	78.05 ± 21.55	83.33 (33.33) *	66.67–100	0.0299
	Emotional	55.97 ± 28.49	58.33 (33.33) *	41.67–75	64.84 ± 26.84	66.67 (41.67) *	41.67–83.33	0.1263
	Social	45.27 ± 31.36	33.33 (50) *	16.67–66.67	56.5 ± 29.32	66.67 (50) *	33.33–83.33	0.0790
Global health status		41.79 ± 19.81	41.67 (16.67) *	33.33–50	49.39 ± 23.15	50 (33.33) *	33.33–66.67	0.0632
Symptom scales	Fatigue	53.23 ± 28.59	44.44 (44.44) *	33.33–77.78	40.65 ± 28.83	33.33 (33.33) *	22.22–55.56	0.0292
	Pain	48.01 ± 35.71	50 (66.67) *	16.67–83.33	34.55 ± 30.59	33.33 (16.67) *	16.67–33.33	0.0707
	Vomiting/nausea	13.18 ± 19.58	0 (16.67) *	0–16.67	5.69 ± 10.94	0 (16.67) *	0–16.67	0.1194
Single-item measures	Dyspnea	22.89 ± 31.36	0 (33.33) *	0–33.33	13.01 ± 24.58	0 (33.33) *	0–33.33	0.1676
	Insomnia	48.76 ± 31.42	33.33 (33.33) *	33.33–66.67	42.28 ± 37.29	33.33 (66.67) *	0–66.67	0.3051
	Appetite loss	33.33 ± 33.83	33.33 (66.67) *	0–66.67	17.07 ± 24.86	0 (33.33) *	0–33.33	0.0181
	Constipation	25.37 ± 31.3	0 (33.33) *	0–33.33	22.76 ± 31.13	0 (33.33) *	0–33.33	0.6440
	Diarrhea	21.39 ± 32.15	0 (33.33) *	0–33.33	12.2 ± 26.62	0 (0) *	0–0	0.1454
	Financial difficulties	38.81 ± 35.1	33.33 (66.67) *	0–66.67	30.08 ± 35.59	33.33 (33.33) *	0–33.33	0.1705

* Non-distribution based on the Shapiro–Wilk test (*p* < 0.05); Group 1—individuals who reported consulting a dietitian; Group 2—individuals who did not report consulting a dietitian.

**Table 7:** Results of the five functional scales, global quality of life scale, three symptom scales, and six individual symptom scales of the EORTC QLQ-C30, stratified by having declared to solicit nutritional information.

Measures	Subdomain	Group 1 (*n* = 71)			Group 2 (*n* = 37)			*p*-Value
		Mean Score ± SD	Median (IQR)	95%CI	Mean Score ± SD	Median (IQR)	95%CI	
Functional scales	Physical	61.31 ± 27.94	60 (46.67) *	40–86.67	73.69 ± 20.06	80 (40) *	53.33–93.33	0.0354
	Role	50.47 ± 34.15	50 (50) *	16.67–66.67	67.57 ± 32.38	66.67 (50) *	50–100	0.0127
	Cognitive	63.85 ± 28.73	66.67 (50) *	33.33–83.33	80.63 ± 22.05	83.33 (33.33) *	66.67–100	0.0027
	Emotional	54.11 ± 28.31	58.33 (41.67) *	33.33–75	69.37 ± 25.08	75 (50) *	41.67–91.67	0.0029
	Social	43.19 ± 30.42	33.33 (50) *	16.67–66.67	61.71 ± 28.56	66.67 (50) *	33.33–83.33	0.0025
Global health status		40.96 ± 19.3	33.33 (25) *	25–50	25.23 ± 27.67	33.33 (33.33)	0–33.33	0.0161
Symptom scales	Fatigue	55.09 ± 29.6	55.56 (44.44) *	33.33–77.78	30.63 ± 32.04	33.33 (50) *	0–50	0.0006
	Pain	49.30 ± 33.97	33.33 (66.67) *	16.67–83.33	8.56 ± 17.4	0 (16.67) *	0–16.67	0.0031
	Vomiting/nausea	11.27 ± 17.08	0 (16.67) *	0–16.67	18.92 ± 30.97	0 (33.33) *	0–33.33	0.4523
Single-item measures	Dyspnea	19.25 ± 28.54	0 (33.33) *	0–33.33	36.94 ± 31.21	33.33 (66.67) *	0–66.67	0.7441
	Insomnia	51.17 ± 34.19	66.67 (33.33) *	33.33–66.67	31.53 ± 36.81	33.33 (66.67) *	0–66.67	0.0277
	Appetite loss	24.88 ± 28.57	33.33 (33.33) *	0–33.33	20.72 ± 28.71	0 (33.33) *	0–33.33	0.6421
	Constipation	26.29 ± 32.33	0 (33.33) *	0–33.33	11.71 ± 22.52	0 (33.33) *	0–33.33	0.5225
	Diarrhea	21.13 ± 33.45	0 (33.33) *	0–33.33	25.23 ± 27.67	33.33 (33.33) *	0–33.33	0.3717
	Financial difficulties	40.85 ± 37.88	33.33 (66.67) *	0–66.67	30.63 ± 32.04	33.33 (50) *	0–50	0.0502

* Non-distribution based on the Shapiro–Wilk test (*p* < 0.05); Group 1—individuals who soliciting nutritional information; Group 2—individuals who did not solicit nutritional information.

**Table 8:** Stepwise multiple regression analysis for QoL (global health status) in a model including five functional scales (physical, role, cognitive, emotional, and social) and three symptom scales (fatigue, pain, and nausea/vomiting) as variables.

	Unstandardized Coefficients		Standardized Coefficients β	*p*
	β	SE		
Constant			11.7007	0.0181
Functional scales—physical functioning	0.4909	0.0744	0.4018	<0.0001
Functional scales—social functioning	0.2788	0.0765	0.1925	0.0004
Appetite loss	−0.1580	0.0735	−0.10673	0.0340

SE—standard error.

## Data Availability

The data presented in this study are available on request from the corresponding author. The data are not publicly available due to ethical restrictions.

## Supplementary Materials

The following supporting information can be downloaded at https://www.mdpi.com/article/10.3390/nu16183207/s1, Table S1: Results of the five functional scales, global quality of life scale, three symptom scales, and six individual symptom scales of the EORTC QLQ-C30, stratified by level of education; Table S2: Results of the five functional scales, global quality of life scale, three symptom scales, and six individual symptom scales of the EORTC QLQ-C30, stratified by place of residence; Table S3: Results of stepwise multiple regression analysis for sum on scale of EORTC QLQ-C30 (*n* = 108); Table S4: Results of stepwise multiple regression analysis for role domain on scale of EORTC QLQ-C30 (*n* = 108); Table S5: Results of stepwise multiple regression analysis for physical domain on the scale of EORTC QLQ-C30 (*n* = 108); Table S6: Results of stepwise multiple regression analysis for cognitive domain of scale of EORTC QLQ-C30 (*n* = 108); Table S7: Results of stepwise multiple regression analysis for emotional domain of scale of EORTC QLQ-C30 (*n* = 108); Table S8: Results of stepwise multiple regression analysis for emotional domain of scale of EORTC QLQ-C30 (*n* = 108); Table S9: Results of stepwise multiple regression analysis for social domain of scale of EORTC QLQ-C30 (*n* = 108); Table S10: Results of stepwise multiple regression analysis for fatigue domain of scale of EORTC QLQ-C30 (*n* = 108); Table S11: Results of stepwise multiple regression analysis for pain domain of scale of EORTC QLQ-C30 (*n* = 108); Table S12: Results of stepwise multiple regression analysis for vomiting/nausea domain of scale of EORTC QLQ-C30 (*n* = 108); Table S13: Results of stepwise multiple regression analysis for dyspnea domain of scale of EORTC QLQ-C30 (*n* = 108); Table S14: Results of stepwise multiple regression analysis for insomnia domain of scale of EORTC QLQ-C30 (*n* = 108); Table S15: Results of stepwise multiple regression analysis for appetite loss domain of scale of EORTC QLQ-C30 (*n* = 108); Table S16: Results of stepwise multiple regression analysis for constipation domain of scale of EORTC QLQ-C30 (*n* = 108); Table S17: Results of stepwise multiple regression analysis for diarrhea domain of scale of EORTC QLQ-C30 (*n* = 108); Table S18: Results of stepwise multiple regression analysis for financial difficulties domain of scale of EORTC QLQ-C30 (*n* = 108); Table S19: Results of stepwise multiple regression analysis for global health status (QoL) of EORTC QLQ-C30 (*n* = 108).
