# Supplementary material for: Multifactorial Analysis of Influences on Quality of Life in Cancer Patients
Source: Nutrients. 2024 Sep 22;16(18):3207. doi: 10.3390/nu16183207 (PMC11435082; doi:10.3390/nu16183207)
Supplement: Supplementary file 1 [file nutrients-16-03207-s001.zip › nutrients-3201721-supplementary.pdf]

nutrients

MDPI

Supplemantary

Supplementary Table S1. Results of the five functional scales, a global quality of life scale, three symptom scales, and six individual symptom scales of the EORTC QLQ-C30, stratified by level of education.

Measures	Sub-domain	Group 1 (*n*=49)			Group 2 (*n*=59)			p-Value	
		Means score ± SD	Median (IQR)	95%CI	Means score ± SD	Median (IQR)	95%CI		
Functional Scales	Physical	59.32±27.56	60 (46.67)*	40-86.67	70.73±23.81	80 (33.33)*	53.33-86.67	0.0347	
	Role	52.04±33.62	66.67 (33.33)*	33.33-66.67	59.89±34.89	66.67 (66.67)*	33.33-100	0.2123	
	Cognitive	62.24±29.02	66.67 (50)*	33.33-83.33	75.71±25.2	83.33 (33.33)*	66.67-100	0.0148	
	Emotional	56.63±31.18	58.33 (41.67)*	33.33-75	61.58±25.29	66.67 (41.67)	41.67-83.33	0.5270	
	Social	45.92±32.9	50 (50)*	16.67-66.67	52.54±29.17	50 (33.33)*	33.33-66.67	0.2944	
Global health status		41.67±19.91	41.67 (25)	25-50	47.18±22.35	50 (33.33)	33.33-66.67	0.1833	0.0306
Symptom Scales	Fatigue	54.65±31.33	55.56 (44.44)*	33.33-77.78	43.31±26.48	33.33 (33.33)*	22.22-55.56	0.0306	
	Pain	44.22±34.95	33.33 (66.67)*	16.67-83.33	41.81±34.1	33.33 (50)*	16.67-66.67	0.7442	
	Vomiting/nausea	10.54±17.24	0 (16.67)*	0-16.67	10.17±17.24	0 (16.67)*	0-16.67	0.9739	
Single-item measures	Dyspnoea	23.81±33.33	0 (33.33)*	0-33.33	15.25±25.01	0 (33.33)*	0-33.33	0.1987	
	Insomnia	43.54±33.47	33.33 (33.33)*	33.33-66.67	48.59±34.08	33.33 (33.33)*	33.33-66.67	0.4416	
	Appetite loss	23.13±28.22	0 (33.33)*	0-33.33	30.51±34.07	33.33 (33.33)*	0-33.33	0.3162	
	Constipation	25.17±30.07	0 (33.33)*	0-33.33	23.73±32.19	0 (33.33)*	0-33.33	0.6430	
	Diarrhea	23.13±34.83	0 (33.33)*	0-33.33	13.56±25.61	0 (33.33)*	0-33.33	0.1510	
	Financial difficulties	35.37±38.13	33.33 (66.67)*	0-66.67	35.59±33.26	33.33 (66.67)*	0-66.67	0.7132	

* The non-distribution based on the Shapiro-Wilk test ($p<0.05$); Group 1 – individuals with higher education level; Group 2 – without higher education level.

Supplementary Table S2. Results of the five functional scales, a global quality of life scale, three symptom scales, and six individual symptom scales of the EORTC QLQ-C30, stratified by place of residence.

Measures	Sub-domain	Group 1 (*n*=58)			Group 2 (*n*=50)			p-Value	
		Means score ± SD	Median (IQR)	95%CI	Means score ± SD	Median (IQR)	95%CI		
Functional Scales	Physical	63.91±25.81	63.33 (40)*	46.67-86.67	67.47±26.55	73.33 (40)*	46.67-86.67	0.4556	
	Role	56.32±34.17	66.67 (50)*	33.33-83.33	56.33±34.97	66.67 (50)*	33.33-83.33	0.9550	
	Cognitive	69.83±26.57	75 (50)*	50-100	69.33±29.23	75 (50)*	50-100	0.9297	
	Emotional	59.91±29.88	62.5 (41.67)*	41.67-83.33	58.67±26.13	66.67 (33.33)*	41.67-75	0.7056	
	Social	48.85±31.2	50 (33.33)*	33.33-66.67	50.33±30.95	50 (33.33)	33.33-66.67	0.7833	
Global health status		42.82±18.7	41.67 (25)	33.33-58.33	46.83±24.1	50 (33.33)	33.33-66.67	0.3321	0.1272
Symptom Scales	Fatigue	51.72±27.18	50 (33.33)*	33.33-66.67	44.67±31.23	33.33 (44.44)*	22.22-66.67	0.1272	
	Pain	40.23±32.9	33.33 (50)*	16.67-66.67	46±36.04	33.33 (66.67)*	16.67-83.33	0.3976	
	Vomiting/nausea	10.34±17.61	0 (16.67)*	0-16.67	10.33±16.8	0 (16.67)*	0-16.67	0.9104	
Single-item measures	Dyspnoea	21.26±29.75	0 (33.33)*	0-33.33	16.67±28.77	0 (33.33)*	0-33.33	0.3225	
	Insomnia	39.08±29.38	33.33 (33.33)*	33.33-66.67	54.67±36.74	66.67 (66.67)*	33.33-100	0.0212	
	Appetite loss	25.29±30.15	16.67 (33.33)*	0-33.33	29.33±33.43	33.33 (33.33)*	0-33.33	0.5719	

Constipation	26.44±32.9	0 (66.67)*	0-66.67	22.0±29.05	0 (33.33)*	0-33.33	0.5699
Diarrhea	12.07±22.25	0 (33.33)*	0-33.33	24.67±36.77	0 (33.33)*	0-33.33	0.1186
Financial difficulties	33.33±33.62	33.33 (66.67)*	0-66.67	38±37.5	33.33 (66.67)*	0-66.67	0.6011

* The non-distribution based on the Shapiro-Wilk test (p<0.05); Group 1 – individuals living in big city and large town (above 20,000 inhabitants); Group 2 – individuals living in village and small town (up to 20,000 inhabitants).

Supplementary Table S3. Results of the stepwise multiple regression analysis for Sum of scale of EORTC QLQ-C30 (n=108).

	Unstandardized coefficients		Standardized coefficients β	*p*
	β	SE		
Constant			87.1655	<0.0001
Reduced food consumption	-0.2241	0.0909	-6.5662	0.0153
Patients soliciting nutritional information	-0.2948	0.0909	-9.3159	0.0016

SE – standard error.

Supplementary Table S4. Results of the stepwise multiple regression analysis for Role domain of scale of EORTC QLQ-C30 (n=108).

	Unstandardized coefficients		Standardized coefficients β	*p*
	β	SE		
Constant			58.8621	<0.0001
Types of nutrition support	-0.2477	0.0921	-19.3464	0.0083
Patients soliciting nutritional information	0.2250	0.0921	16.7186	0.0162

SE – standard error.

Supplementary Table S5. Results of the stepwise multiple regression analysis for Physical domain of scale of EORTC QLQ-C30 (n=108).

	Unstandardized coefficients		Standardized coefficients β	*p*
	β	SE		
Constant			67.4894	<0.0001
Types of nutrition support	-0.215328	0.096654	-12.7611	0.0280
Education level	0.246379	0.096654	7.9930	0.0122

SE – standard error.

Supplementary Table S6. Results of the stepwise multiple regression analysis for Cognitive domain of scale of EORTC QLQ-C30 (n=108).

	Unstandardized coefficients		Standardized coefficients β	*p*
	β	SE		

Constant			26.1354	0.0137
Seeking consultation with a dietitian	0.2395	0.0899	13.6086	0.0090
Gender	0.3051	0.0899	16.8757	0.0010

SE – standard error.

Supplementary Table S7. Results of the stepwise multiple regression analysis for Emotional domain of scale of EORTC QLQ-C30 (n=108).

	**Unstandardized coefficients**		**Standardized coefficients β**	***p***
	β	SE		
Constant			1.1593	0.9232
Type of cancer	0.2150	0.0919	6.8570	0.0213
Gender	0.3903	0.0908	21.8807	0.0004
Employment	0.2372	0.0888	3.5552	0.0088

SE – standard error.

Supplementary Table S8. Results of the stepwise multiple regression analysis for Emotional domain of scale of EORTC QLQ-C30 (n=108).

	**Unstandardized coefficients**		**Standardized coefficients β**	***p***
	β	SE		
Constant			44.4341	0.0002
Types of nutrition support	-0.2139	0.0916	-15.0374	0.0214
Seeking consultation with a dietitian	0.2753	0.0916	18.4128	0.0033

SE – standard error.

Supplementary Table S9. Results of the stepwise multiple regression analysis for Social domain of scale of EORTC QLQ-C30 (n=108).

	**Unstandardized coefficients**		**Standardized coefficients β**	***p***
	β	SE		
Constant			25.8788	0.0035
Patients soliciting nutritional information	0.2710	0.0935	18.1212	0.0046

SE – standard error.

Supplementary Table S10. Results of the stepwise multiple regression analysis for Fatigue domain of scale of EORTC QLQ-C30 (n=108).

	**Unstandardized coefficients**		**Standardized coefficients β**	***p***
	β	SE		
Constant			107.9809	<0.0001

Patients soliciting nutritional information	-0.2297	0.0929	-14.4949	0.0150
Gender	-0.2152	0.0922	-12.5436	0.0215
Age	-0.1883	0.0910	-0.3835	0.0411

SE – standard error.

Supplementary Table S11. Results of the stepwise multiple regression analysis for Pain domain of scale of EORTC QLQ-C30 (n=108).

	Unstandardized coefficients		Standardized coefficients β	*p*
	β	SE		
Constant			85.2426	<0.0001
Reduced food consumption	-0.2109	0.0931	-14.5068	0.0256
Patients soliciting nutritional information	-0.2207	0.0931	-16.3816	0.0196

SE – standard error.

Supplementary Table S12. Results of the stepwise multiple regression analysis for Vomiting/nausea domain of scale of EORTC QLQ-C30 (n=108).

	Unstandardized coefficients		Standardized coefficients β	*p*
	β	SE		
Constant			20.6771	<0.0001
Seeking consultation with a dietitian	-0.2129	0.0949	-7.4930	0.0270

SE – standard error.

Supplementary Table S13. Results of the stepwise multiple regression analysis for dyspnoea domain of scale of EORTC QLQ-C30 (n=108).

	Unstandardized coefficients		Standardized coefficients β	*p*
	β	SE		
Constant			34.5833	<0.0001
Reduced food consumption	-0.1825	0.0955	-10.6944	0.0491

SE – standard error.

Supplementary Table S14. Results of the stepwise multiple regression analysis for Insomnia domain of scale of EORTC QLQ-C30 (n=108).

	Unstandardized coefficients		Standardized coefficients β	*p*
	β	SE		
Constant			111.3361	<0.0001

Reduced food consumption	-0.2259	0.0906	-15.2714	0.0142
Age	-0.2240	0.0905	-0.5274	0.0150
Place of residence	-0.2078	0.0909	-4.1407	0.0242

SE – standard error.

Supplementary Table S15. Results of the stepwise multiple regression analysis for Appetite loss domain of scale of EORTC QLQ-C30 (n=108).

	Unstandardized coefficients		Standardized coefficients β	*p*
	β	SE		
Constant			28.9776	0.0130
Types of nutrition support	0.3403	0.0842	24.4404	0.0001
Reduced food consumption	-0.3562	0.0842	-22.5650	<0.0001

SE – standard error.

Supplementary Table S16. Results of the stepwise multiple regression analysis for Constipation domain of scale of EORTC QLQ-C30 (n=108).

	Unstandardized coefficients		Standardized coefficients β	*p*
	β	SE		
Constant			85.9814	0.0001
Weight loss	-0.3428	0.1283	-22.5172	0.0087
Over 10% weight loss	-0.3377	0.1283	-20.9213	0.0097

SE – standard error.

Supplementary Table S17. Results of the stepwise multiple regression analysis for Diarrhea domain of scale of EORTC QLQ-C30 (n=108).

	Unstandardized coefficients		Standardized coefficients β	*p*
	β	SE		
Constant			74.4516	<0.0001
Age	-0.2127	0.0935	-0.4507	0.0249
Education level	-0.2217	0.1016	-8.3746	0.0312
Place of residence	-0.2907	0.0999	-5.2130	0.0044

SE – standard error.

Supplementary Table S18. Results of the stepwise multiple regression analysis for Financial difficulties domain of scale of EORTC QLQ-C30 (n=108).

	Unstandardized coefficients		Standardized coefficients β	*p*
	β	SE		
Constant			39.8516	0.0008
Patients soliciting nutritional information	-0.2135	0.0905	-16.3517	0.0202
BMI	-0.2177	0.0909	-0.2618	0.0184
Economic situation	0.3614	0.0906	10.0046	0.0001

SE – standard error; BMI – Body mass index.

Supplementary Table S19. Results of the stepwise multiple regression analysis for Global Health Status (QoL) of EORTC QLQ-C30 (n=108).

	Unstandardized coefficients		Standardized coefficients β	*p*
	β	SE		
Constant			17.7611	0.0856
Types of nutrition support	-0.1930	0.0878	-9.3647	0.0301
Reduced food consumption	0.1857	0.0889	7.9463	0.0391
Patients soliciting nutritional information	0.2156	0.0912	9.9523	0.02000
Gender	0.2282	0.0921	9.731	0.0149

SE – standard error.
